# Identification of dynamic driving styles based on behavioral primitives

**DOI:** 10.1038/s41598-026-38787-y

**Published:** 2026-03-26

**Authors:** Xuelian Zheng, Wenyu Kang, Yuanyuan Ren, Xiansheng Li, Jianfeng Xi

**Affiliations:** Transportation College, Jilin University, Changchun, 130012 China

**Keywords:** Dynamic driving style, Behavioral primitive, Primitive risk index, Primitives transition risk index, The dynamic driving style evaluation model, Style classification, Applied mathematics, Computer science, Scientific data

## Abstract

Driving style reflects drivers’ vehicle manipulation and driving habits, it is essential for understanding and analyzing drivers’ dynamic behavior and decision-making process. To better understand the intrinsic characteristics of how drivers make decisions and control vehicles based on external conditions, this paper proposes an unsupervised framework for dynamic driving style classification based on behavior primitives. The framework consists of three key stages: primitive extraction, model development, and dynamic driving style classification. Unsupervised techniques are employed to extract meaningful behavior primitives from time-series driving data. The primitive serves as the fundamental unit for dynamic driving style analysis, and a driving style evaluation model is built using a linear weighting approach. This model quantifies the risks of primitives and the transition risks between adjacent primitives. The thresholds for classifying cautious, average, and aggressive styles are determined using an improved particle swarm optimization algorithm. The proposed dynamic driving style classification framework comprehensively considers the characteristics of the current primitive and its surrounding context. By preserving the temporal features of driving behavior, the framework provides fine-grained classification of dynamic driving styles. Additionally, a deeper understanding of drivers’ dynamic driving style and their long-term driving behavior can be gained based on this framework, which will benefit the development of AVs and ADASs.

## Introduction

 Driving behavior refers to the vehicle driving decisions and operations made by driver in response to real-time traffic conditions. It reflects the driver’s unique behavior patterns and is of significant importance for driver behavior prediction^1,2^, traffic safety analysis^3^, and the development of autonomous driving technology^4–6^. As a higher-level representation of driving behavior, driving style reflects individual’s decision-making habits^7^. It helps reveal the driver’s behavior patterns and provides data support for the design of personalized driving assistance systems^8^.

Currently, research on driving styles predominantly focuses on holistic concepts, offering broad and qualitative definitions of driving behaviors. Common approaches in this area include questionnaire surveys and objective driving data analysis. While the questionnaire survey method is convenient, its results are heavily influenced by subjective judgement, limiting its validity^9,10^. On the other hand, objective driving data analysis relies on data-driven approaches that directly capture the characteristics of driving styles. Typically, statistical analysis is performed on driving behavior data sequences, converting them into discrete statistical features. These features are then used to generate offline, generalized descriptions of driving styles^11–13^. The current research has mostly centered on steady-state characteristics, such as long-term average speed and the total frequency of harsh acceleration events, often categorizing driving styles into broad static classifications like aggressive or conservative. This approach overlooks the adaptability of driving styles to specific scenarios and fails to account for microscopic dynamic features.

Driving style, which refers to the characteristic behavior of a driver when operating a vehicle in real-world conditions, can vary depending on factors such as time periods, environmental conditions, and traffic challenges^7,14^. While a holistic driving style can only capture a driver’s long-term stable behavioral preferences, it fails to account for short-term fluctuations in driving behavior^14–16^. Research into dynamic driving styles allows for a more detailed analysis of the relationship between external factors and driver responses. This approach significantly enhances the accuracy of driving style evaluations, contributing to improved traffic safety, an optimized driving experience, and the advancement of autonomous driving technology.

To achieve a more nuanced understanding of driving styles, scholars have developed new methods to facilitate the detailed identification of driving behaviors. Using 8-second intervals and specific driving scenarios as analysis units, unsupervised clustering techniques based on statistical features are employed to recognize driving styles within each cycle^17,18^. Some researchers have divided multi-dimensional driving behavior data into meaningful behavioral primitives^19–25^. By analyzing the distribution of these primitives^23^, the frequency of high-risk behavior occurrences and the transition probabilities between different behavioral primitives^19–21,24^, driving styles are classified into categories such as cautious, normal, and aggressive. Existing studies have made significant progress in refining driving style analysis by introducing local driving style evaluation units, and the dynamic driving features are revealed to some extent. However, most of the current research focuses on local behavioral characteristics within discrete time segments, without thoroughly accounting for the dynamic evolution of driving behavior over time. This leads to a gap in the temporal continuity of style representation. Therefore, the core focus of this research is to model dynamic driving styles based on the temporal characteristics of driving behavior.

This paper employs behavioral primitives as the fundamental unit for dynamic driving style evaluation. A dynamic evaluation model is constructed by quantifying the risks associated with both the current primitive and primitive transitions. The model utilizes a weighted summation method, incorporating three key components: the inherent risk of the current primitive, the risk of transitioning from the previous to the current primitive, and the risk of transitioning from the current to the next primitive. While the risk of a primitive is assessed based on its specific risk characteristics, the transition risk index is formulated by considering the risk differential before and after the transition, the risk of the succeeding primitive, and the probability of the transition. Finally, following the calculation of the driving style score, the Particle Swarm Optimization (PSO) algorithm is applied to determine the thresholds for classifying cautious, normal, and aggressive driving styles.

The structure of this paper is as follows: Sect. 2 uses unsupervised methods to obtain driving behavior primitives; Sect. 3 introduces the dynamic driving style assessment framework in function of primitives; Sect. 4 constructs a dynamic driving style evaluation model and uses the Particle Swarm Optimization (PSO) algorithm to obtain thresholds for cautious, general, and aggressive driving styles; Sect. 5 analyzes and discusses the dynamic driving styles of 17 drivers.

## Driving behavioral primitives

### Definition and extraction

Driving behavior primitives are the smallest segments of driving behavior data with clear physical meaning. Within a primitive, vehicle operational state either remains constant or changes at a constant speed. The types of driving behavior primitives are limited, and driver selects different primitives based on the driving environment.

The extraction of driving behavior primitives typically involves two key steps: time-series segmentation and segment clustering. Segmentation aims to derive the sequential order of primitives from driving behavior data, while clustering focuses on identifying the categories and physical meanings of these segments. In this study, natural driving data was collected from 17 participants via a driving simulator experiment, accumulating approximately 4.82 h of data. The experimental route comprised 11 curves and covered a round-trip distance of approximately 21.7 km.

To ensure a consistent baseline for comparative analysis, the experiment was conducted in a controlled environment. This setup included sparse surrounding traffic and pre-scripted traffic events triggered under specific conditions. The data collected in this setting implicitly captures both the drivers’ general preferences and their response characteristics to unexpected events, thereby providing a solid foundation for the comparative analysis of driving styles.

**Fig. 1:**
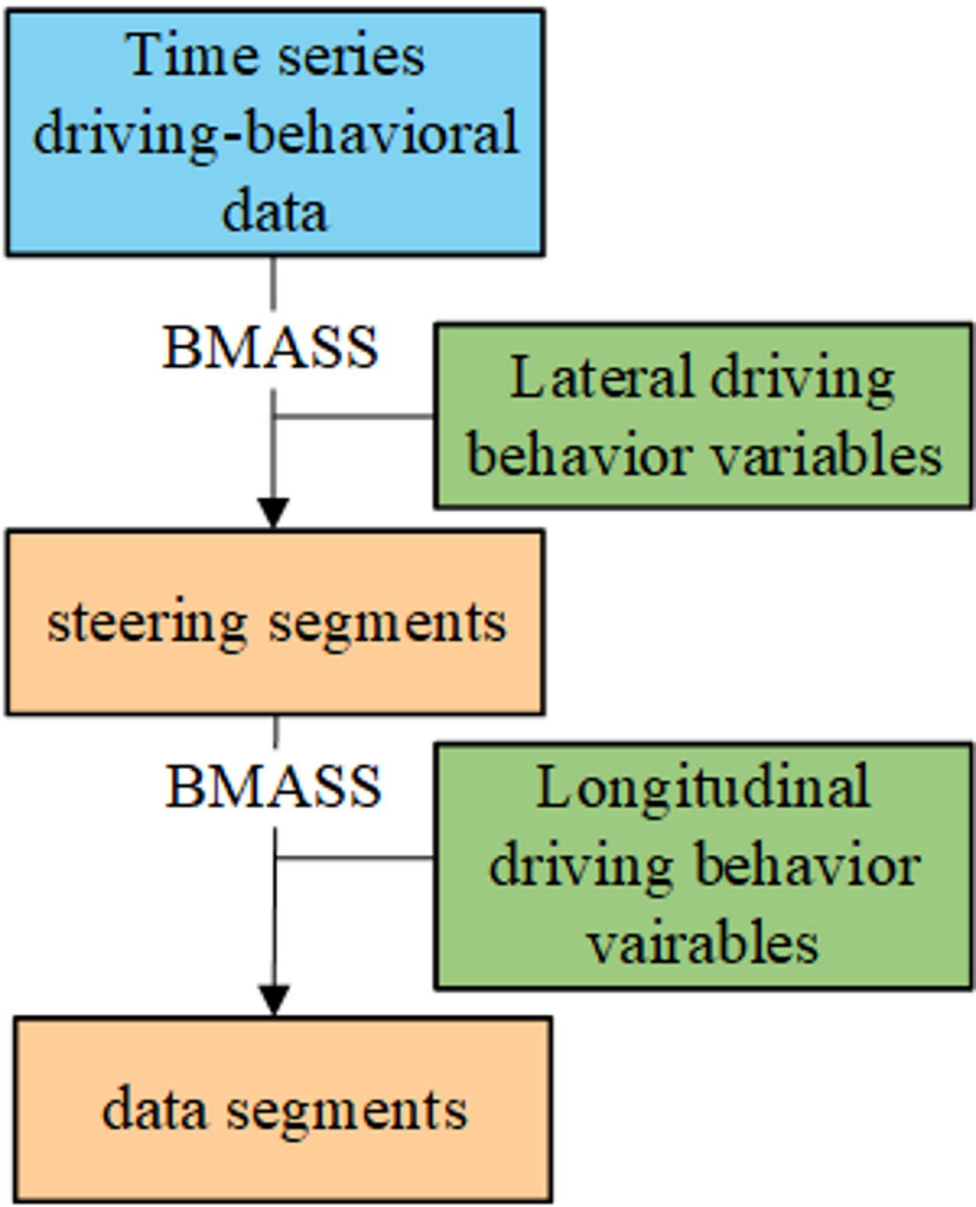
Driving-behavior segmentation based on Hierarchical BMASS structure. The green box represents the input feature variables, while the orange box represents the time-series segmentation results.

While the conventional BMASS method is capable of handling multidimensional variables, it generally assumes that the input dimensions are highly correlated. However, driving behavior data presents a unique challenge: lateral and longitudinal motions are physically decoupled and exhibit significantly different variation magnitudes. When lateral and longitudinal variables are fed simultaneously into the conventional BMASS, the algorithm tends to prioritize the dimension with the dominant variation, thereby masking the subtler yet physically critical variations in the other dimension. Consequently, the segmentation results reflect only one dimension of variation and fail to capture the actual driving situation comprehensively. To overcome this limitation, a dual-layer hierarchical BMASS (H-BMASS) method is employed for data segmentation to identify segment boundaries that more accurately reflect actual driving conditions, as illustrated in Fig. 1^26^. The method establishes a hierarchical processing order for input features based on their physical interpretability and the correlation of their variation trends. Specifically, driving behavior features are categorized into lateral and longitudinal dimensions. The H-BMASS initially performs a coarse segmentation based on lateral features. Lateral acceleration and vehicle yaw angle serve as inputs to extract steering behavior primitives, resulting in a total of 840 steering segments. Subsequently, longitudinal features are applied to refine these steering segments. Longitudinal speed and longitudinal acceleration are used for a second round of segmentation, effectively decomposing the initial steering segments based on speed variations. Ultimately, this process yielded a total of 2,763 driving behavior primitives.

### Clustering and semantic analysis

The LDA method is used to cluster the 2763 driving behavior primitives. As a bag-of-words model, LDA processes discretized numerical data and fails to capture the temporal characteristics inherent in time-series data. To accurately cluster driving behavior segments, it is essential to account for the temporal features, as ignoring these aspects may cause the model to miss subtle differences between segments, especially when they share similar distributions but have distinct change trends. To address this limitation, the driving behavior data is discretized by considering both the data distribution and the change trends over time. Consequently, each behavior data point is represented by several factors: the velocity distribution, velocity change trend, longitudinal acceleration distribution, longitudinal acceleration change trend, steering behavior distribution, lateral acceleration change trend, and yaw angle change trend. This approach allows the model to better capture the dynamics of driving behavior.

The 2,763 primitives are clustered into five types, the statistical feature for each primitive type and its semantic definition are listed in Table 1.

**Table 1:** Statistical feature of the 5 primitive types.

parameters	statistical feature	Primitive type				
		the 1 st type	the 2nd type	the 3rd type	the 4th type	the 5th type
velocity (m/s)	mean	15.09	14.47	6.62	21.12	11.95
	std.	2.18	3.11	3.49	3.72	4.58
Long. acc. (m/s2)	mean	0.00	−0.03	−0.12	−0.11	0.15
	std.	0.44	0.44	0.63	0.55	0.45
Lat. Acc. (m/s2)	mean	−0.01	0.44	0.00	−0.23	−0.62
	std.	0.27	0.75	0.12	0.58	0.73
Yaw rate (rad/s)	mean	0.02	0.11	0.03	0.03	0.14
	std.	0.03	0.19	0.13	0.04	0.21

The semantic definition for the 5 primitive types are listed as follows:

- *The 1 st primitive type*: going straight with medium-speed and gradual speed variation;
- *The 2nd primitive type*: turning left with medium-speed and gradual speed variation;
- *The 3rd primitive type*: going straight with low-speed and gradual decelerating;
- *The 4th primitive type*: going straight with high-speed and rapid decelerating;
- *The 5th primitive type*: turning right with medium-speed and rapid acceleration.

## The framework for dynamic driving styles identification using behavioral primitives

**Fig. 2:**
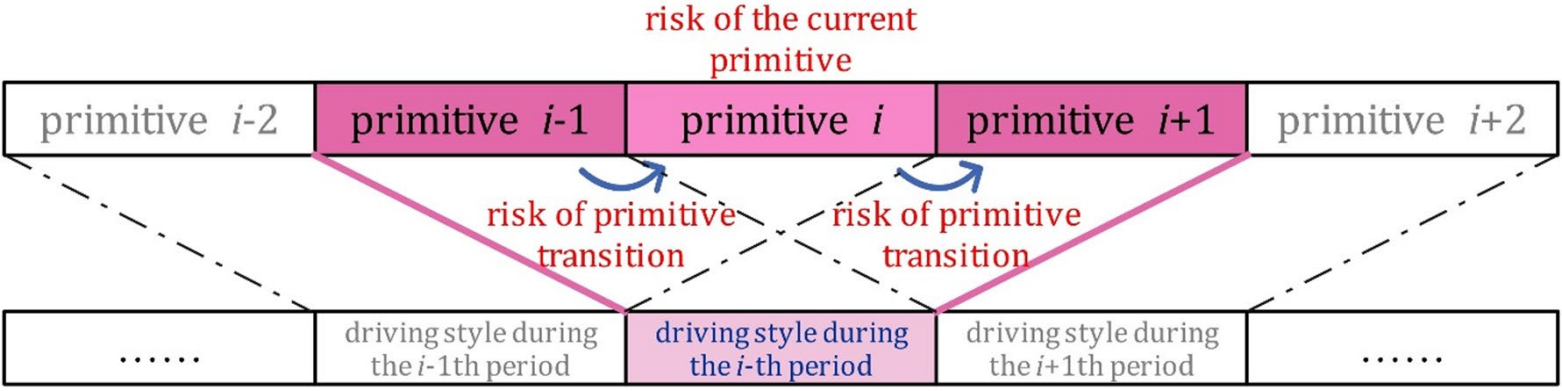
The principle of dynamic driving style classification using the risks of behavioral primitives and the risks of transitions between primitives.

Driving behavior primitives are the smallest data segments that hold physical significance, representing the driver’s perception, understanding, prediction, and the decision-making within the current environment. This characteristic allows primitive to serve as the fundamental units for capturing and analyzing driving style. When examining driving style at the primitive level, it is influenced not only by the intrinsic properties of the primitive, but also by the transition features between the adjacent primitives. As illustrated in Fig. 2, the driving style during the time segment corresponding to primitive *i* is determined by three components: (1) the characteristics of primitive *i*, (2) the transition features from primitive *i-1* to *i*, and (3) the transition features from primitive *i* to *i +* 1.

**Fig. 3:**
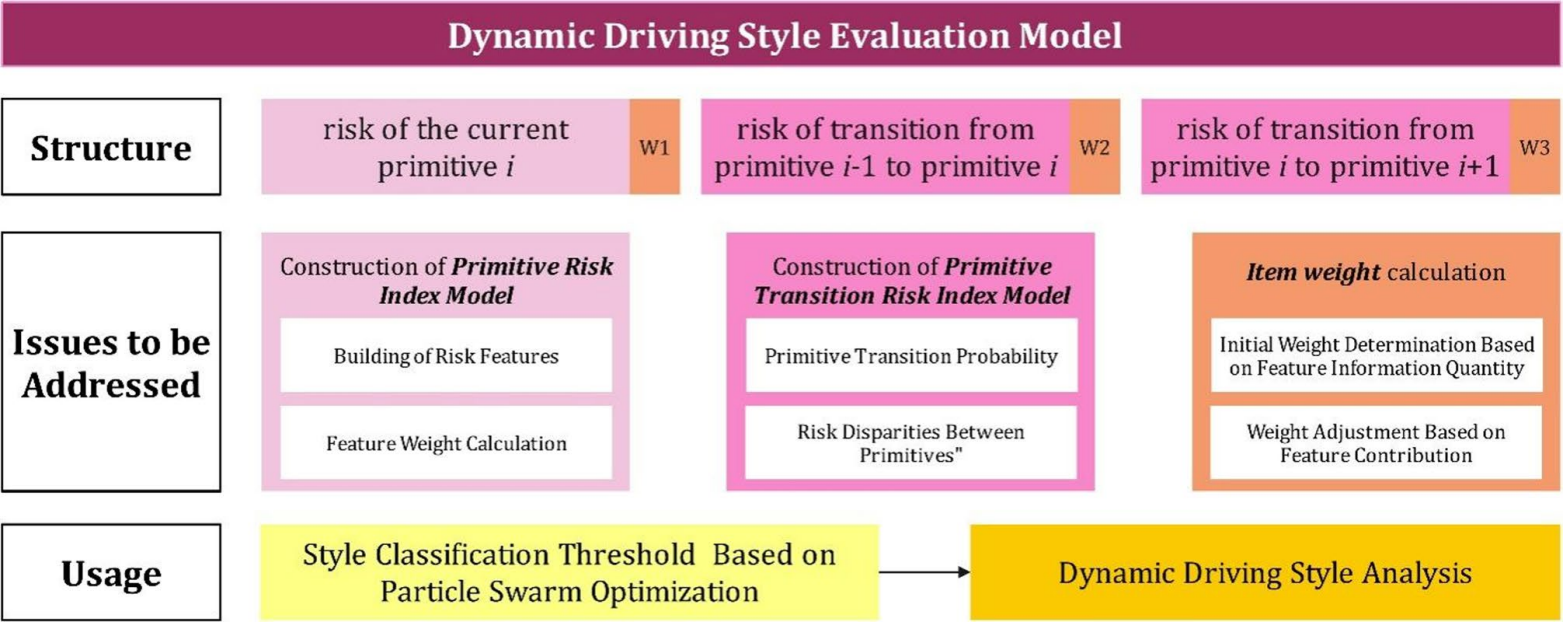
The framework for identifying dynamic driving styles as function of behavioral primitives.

Driving styles are typically classified into cautious, average, and aggressive types based on the evaluation of multidimensional risks during driving. This paper analyzes the dynamic changes in driving styles at the primitive level and constructs a function of dynamic driving style based on primitives. The dynamic driving style evaluation framework is shown in Fig. 3. The risk of the current primitive, the risk of transitioning from the previous primitive to the current one, and the risk of transitioning from the current primitive to the next one together form the driving style evaluation model. Therefore, to obtain a complete dynamic driving style assessment model, the following issues need to be addressed: (1) the expression of the current primitive’s risk; (2) the expression of the risk of transitioning into and out of a primitive, i.e., the expression of primitive transition risk; (3) the allocation of weights among the three risk factors. The evaluation model generates style evaluation scores for each primitive, which are then used by a Particle Swarm Optimization (PSO) algorithm to determine the thresholds for classifying the driving style into cautious, average, or aggressive categories.

It should be emphasized that primitive types, rather than specific individual instances, serve as the benchmark for modeling dynamic driving style and evaluating the three risk indices. This approach is adopted because while the set of primitive types is finite, the number of individual primitives grows continuously with driving duration. Each category defines a specific driving behavior pattern, characterizing the driver’s response to environmental changes. Primitives belonging to the same category share similar temporal variation features and data distributions, despite potential differences in details such as duration or fluctuation amplitude. Essentially, they represent the same underlying behavioral pattern, adjusted only slightly to accommodate environmental variations. Consequently, modeling based on primitive types enables the consideration of transition risks and constrains the solution space, which is critical for robust evaluation.

## Method

### Risk index model for behavioral primitives

Driving behavior primitives represent a multidimensional time series that capture driver’s decisions in the current driving environment. Key statistical indicators, such as the mean and standard deviation of driving speed, longitudinal acceleration, lateral acceleration, and vehicle yaw rate, quantify both the safety of driving behavior and help identify high-risk patterns. This paper utilizes these statistical features to assess the risk associated with different primitives. The eight risk features are used to construct a risk index model for behavioral primitives (covering five primitive types in this paper) through the weighted sum method:

1 $$\:Rp_{k} = \sum \: _{{q = 1}}^{8} w_{{kq}} \cdot Rfn_{{kq}}$$

where *Rp*_*k*_ is risk index for the *k*-th type of primitive; *Rfn*_*kq*_ is the value of the *q*-th risk feature of the *k*-th type of primitive after normalization and *w*_*kq*_ is its weight, *k* = 1, 2, …, 5.

To calculate the risk index of each primitive type, the weights of the features in Eq. (1) must be determined. The correlation coefficient method is an objective weighting approach that measures feature importance to the current primitive category by calculating the correlation coefficient between the risk feature and the primitive category. A larger correlation coefficient indicates a stronger relationship and higher feature importance, resulting in a higher weight. Conversely, a smaller correlation coefficient signifies a weaker relationship and lower feature importance, leading to a reduced weight. This method assigns weights to risk features based on the collected data, unaffected by subjective factors. There hence, the correlation coefficient method is used to obtain the weight coefficients of the risk features for five primitive types, as shown in Table 2.

**Table 2:** Weights of the risk features for the five primitive categories.

	w_1_	w_2_	w_3_	w_4_	w_5_	w_6_	w_7_	w_8_
The 1 st type	0.2393	0.0764	0.0467	0.0714	0.0948	0.2242	0.0090	0.2382
The 2nd type	0.0397	0.0895	0.0186	0.0710	0.2782	0.1533	0.2593	0.0905
The 3rd type	0.4587	0.0850	0.0996	0.0573	0.0546	0.2269	0.0128	0.0051
The 4th type	0.4748	0.1030	0.0950	0.0347	0.1158	0.0467	0.0414	0.0886
The 5th type	0.1126	0.0829	0.1110	0.0385	0.2495	0.1225	0.1841	0.0989

As illustrated in Sect. 2.2, the 1st, 3rd, and 4th types of primitives are straight-line primitives, while the 2nd and 5th types are turning primitives. Table 2 indicates that the weight of driving speed is higher for straight-line primitives, whereas the weight of vehicle posture is higher for turning primitives.

**Fig. 4:**
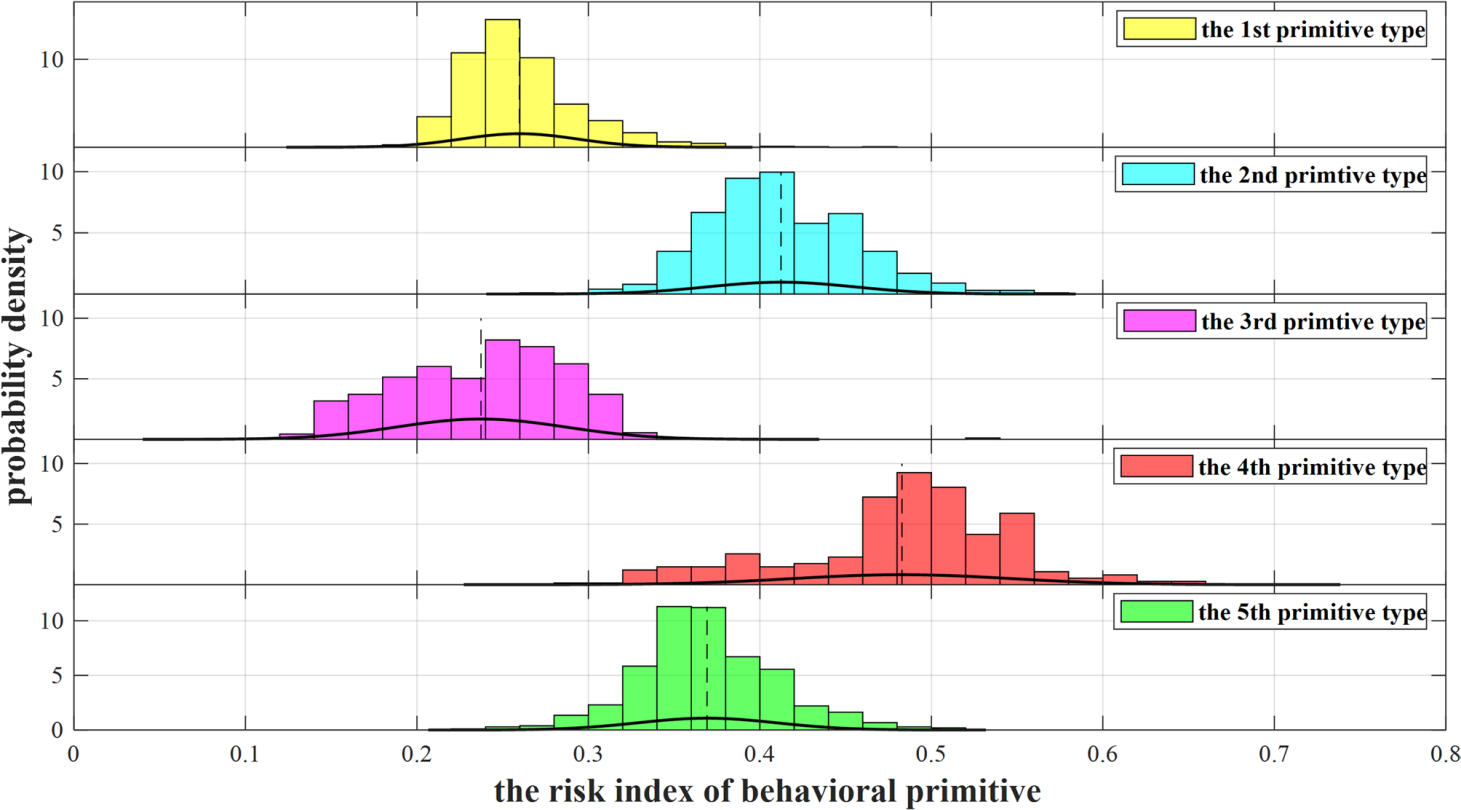
Risk index distribution for the 5 types of primitives.

The probability density distribution of the risk indices for the five primitive types, obtained from Eq. (1), is shown in Fig. 4. There is a low degree of overlap between the five probability density distributions, suggesting the primitive risk index model constructed in this paper is reasonable. Additionally, the risk index distribution for each primitive type is relatively concentrated. Therefore, the average risk index of each primitive type is used as the final quantified result, with values of 0.2598, 0.4123, 0.2373, 0.4829, and 0.3689, respectively.

### Risk index model for primitives transition

Driving behavior is sequential in nature. During the driving process, driver transitions between different behavioral primitive based on the changes in the traffic environment. The transitions reflect driver’s decision-making preferences and serve as an important basis for identifying driving style.

**Fig. 5:**
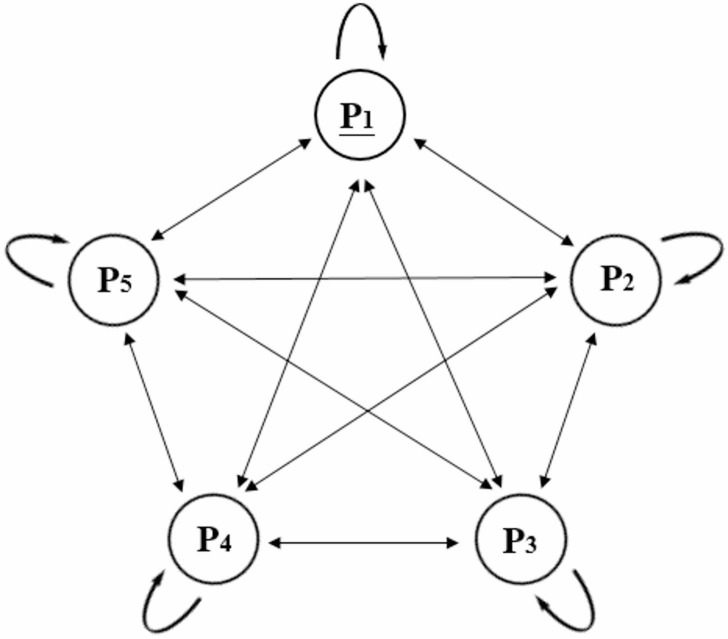
Primitive transition forms.

The transition types between primitives are shown in Fig. 5. Each primitive type can transition to other primitive categories as well as to itself. A transition from *P*_*i*_ to *P*_*j*_ is referred to as a form of primitive transition. There are five primitive types in this paper, theoretically there are 5*5 possible primitive transition forms.

The primitive transition probability can be represented as

2 $$\:{tp}_{ij}=p\left({q}_{k+1}={P}_{j}|{q}_{k}={P}_{i}\right)=p\left({q}_{k+1}={P}_{j},{q}_{k}={P}_{i}\right)/p\left({q}_{k}={P}_{i}\right)$$

Where *q*_*k*_, *q*_*k+1*_ are primitive type at the *k*-th and *k + 1*-th time period; *p*(*q*_*k*+1_=*P*_*j*_, *q*_*k*_=*P*_*i*_) is the joint estimation probability of adjacent primitives; *p*(*q*_*k*_ = *P*_*i*_) is marginal probability of primitive type at the *k*-th time period.

According to the law of large numbers, probability can be estimated using frequency when the sample size is large. Thus, the estimation method for the primitive transition probability is presented by

3 $$\:{tp}_{ij}=n\left({q}_{k+1}={P}_{j},{q}_{k}={P}_{i}\right)/n\left({q}_{k}={P}_{i}\right)$$

Where *tp*_*ij*_∈[0,1], $\:{\sum\:}_{j=1}^{K}{tp}_{ij}=1$, *K* = 5 in this paper. A lower *tp*_*ij*_ indicates a lower occurrence probability for this primitive transition form.

The primitive transition probability matrix *TP*_*K*×*K*_ for the current dataset obtained based on Eq. (3) is shown in Table 3.

**Table 3:** Probability matrix of primitive transition.

	The 1 st type	The 2nd type	The 3rd type	The 4th type	The 5th type
The 1 st type	0.5573	0.1211	0.1068	0.1068	0.1079
The 2nd type	0.3413	0.4850	0.0279	0.0679	0.0778
The 3rd type	0.1264	0.0271	0.6321	0.0045	0.2099
The 4th type	0.1206	0.0992	0.0080	0.5576	0.2145
The 5th type	0.2361	0.1823	0.1152	0.0614	0.4050

A greater difference in primitive risk before and after a transition indicates a higher level of primitive transition risk. Furthermore, the complexity and uncertainty of the incoming primitive provide insight into the potential risks resulting from environmental changes. Therefore, a higher risk of the incoming primitive suggests an increased risk during the transition. Additionally, the transition form also influences the transition risk. If the driver selects a transition form with a low probability, the transition risk is typically higher due to factors such as unfamiliarity with the maneuver, distractions, or inadequate judgment of surrounding traffic conditions. The paper proposes a risk index model for primitive transitions, which incorporates the difference in primitive risk before and after the transition, the risk associated with the incoming primitive, and the probability of transition forms. To effectively quantify the risk differences introduced by various transition forms, a logarithmic transformation will be applied. Thereafter, the risk index of primitive transition can be expressed by

4 $$\:Rp\_T_{{ij}} = Rp_{j} \cdot \:|Rp_{j} - Rp_{i} | \cdot \:\lg \left( {1/tp_{{ij}} } \right)$$

Where *Rp_T*_*ij*_ is the risk index when primitive *i* transits to primitive *j.* 

The risk indices corresponding to the 25 primitive transition forms obtained are shown in Table 4, where the rows represent the preceding primitive and the columns represent the succeeding primitive.

**Table 4:** Risk index matrix of primitive transition.

	The 1 st type	The 2nd type	The 3rd type	The 4th type	The 5th type
The 1 st type	0	0.1328	0.0119	0.2410	0.0897
The 2nd type	0.0426	0	0.1486	0.0917	0.0408
The 3rd type	0.0121	0.2604	0	0.6403	0.0758
The 4th type	0.1226	0.0672	0.2811	0	0.0647
The 5th type	0.0409	0.0304	0.0675	0.1535	0

### The dynamic driving style evaluation

#### The evaluation model

The risk of current primitive reflects the safety of driving actions performed in the current driving environment. High-risk primitives may indicate that the driver’s current driving style is more aggressive, while low-risk primitives suggest that the driver is being more cautious. The risk of primitive transition focuses on the driver’s decision-making ability when road conditions or situations change, reflecting their adaptability and judgment in new environments. Situations with higher primitive transition risks often arise when the driver has insufficient ability to handle new challenges or seeks driving excitement. As the primitive transition risk increases, the driver’s driving style tends to become more aggressive.

The risk associated with the current primitive, the risk of transitioning into the current primitive, and the risk of transitioning out of the current primitive collectively form the feature set for driving style. As these three features have varying levels of influence on driving style, a weighted summation approach is employed to develop the dynamic driving style assessment model

5 $$\:DSS = w_{1} \cdot \:Rpn + w_{2} \cdot \:Rp\_Tin + w_{3} \cdot Rp\_Ton$$

Where *Rpn* is the normalized risk index of current primitive; *Rp_Tin* is the normalized risk index of transitioning into the current primitive; *Rp_Ton* is the normalized risk index of transitioning out of the current primitive; *w*_*1*_, *w*_*2*_, *w*_*3*_ are weights.

#### Weights of the three risk indices components

Given the consideration of the information content of the three items, the entropy weight method is used to calculate their weights for the driving style evaluation model. By the entropy weight method, the initial weights are *w*_*EWM*_ = [0.3260, 0.3356, 0.3384]. The results show that the weights of the three style-related items are similar. The entropy weight method typically only reflects the degree of dispersion of the feature values and cannot capture the correlations between the features. Therefore, the hierarchical analysis method is applied to adjust the model weights.

The traditional Analytic Hierarchy Process (AHP) creates a judgment matrix by evaluating the relative importance of different features based on expert opinions, and assigns weights to these features according to their perceived importance. To minimize subjective bias, the coefficient of variation ratio is employed to quantify the relative importance of the features. The coefficient of variation typically reflects the variability or stability of a feature. A lower coefficient of variation usually indicates greater stability, suggesting that the feature has a smaller influence on driving style, and therefore, its contribution and importance in constructing the style evaluation model are comparatively lower. The judgment matrix constructed using the coefficient of variation ratio is as follows:

$$\:A=\left[\begin{array}{ccc}1&\:{a}_{12}&\:{a}_{13}\\\:1/{a}_{12}&\:1&\:{a}_{23}\\\:1/{a}_{13}&\:1/{a}_{23}&\:1\end{array}\right]$$

Where *a*_*ij*_ is the importance of feature *i* relative to feature *j*. *a*_*ij*_ = *CV*_*i*_/*CV*_*j*_, *CV*_*i*_ is coefficient of variation of feature *i*,* i*, *j* = 1, 2, 3.

The judgment matrix obtained by the dataset used in this paper is as follows:

$$\:A=\left[\begin{array}{ccc}1&\:0.5698&\:0.5703\\\:1.7549&\:1&\:1.0008\\\:1.7534&\:0.9992&\:1\end{array}\right]$$

The matrix shows that the importance of the risk of transitioning into the current primitive is approximately the same as that of the risk of transitioning out of the current primitive. The risk of primitive transition contribute 1.75 times more than the risk of current primitive. A consistency test of the judgment matrix yields a consistency ratio (CR) of 8.54E-16, indicating that the matrix passes the consistency test. The final weight adjustment result of the model is *w*_*AHP*_ = [0.2218, 0.3893, 0.3889]. The results show that the risk of primitive transition contribute the most in the construction of dynamic driving style evaluation model. In contrast, the risk of current primitive has relatively less importance.

The multiplication synthesis method is used to obtain the combined weights, and the dynamic driving style evaluation model is expressed as

6 $$\:w = w_{{EWM}} \cdot \:w_{{AHP}} /\sum \: w_{{EWM}} \cdot w_{{AHP}}$$

7 $$\:DSS = 0.2161 \cdot Rpn + 0.3904 \cdot \:Rp\_Tin + 0.3934 \cdot Rp\_Ton$$

The results indicate that the weights of primitive transition risks are 0.3934 and 0.3904, respectively, highlighting the importance of these two features in identifying dynamic driving styles. This suggests that a driver’s decision-making ability, adaptability in unfamiliar environments, and sustained attention to driving risks in response to changing road conditions directly influence their behavior patterns. If a driver fails to effectively assess surrounding risks during a transition of behavioral primitives or employs low-frequency primitive transition patterns, they may exhibit more aggressive driving behavior. The weight of the current primitive risk is 0.2161. While this weight is relatively lower than that of the transition risk, its contribution to the model should not be underestimated.

**Fig. 6:**
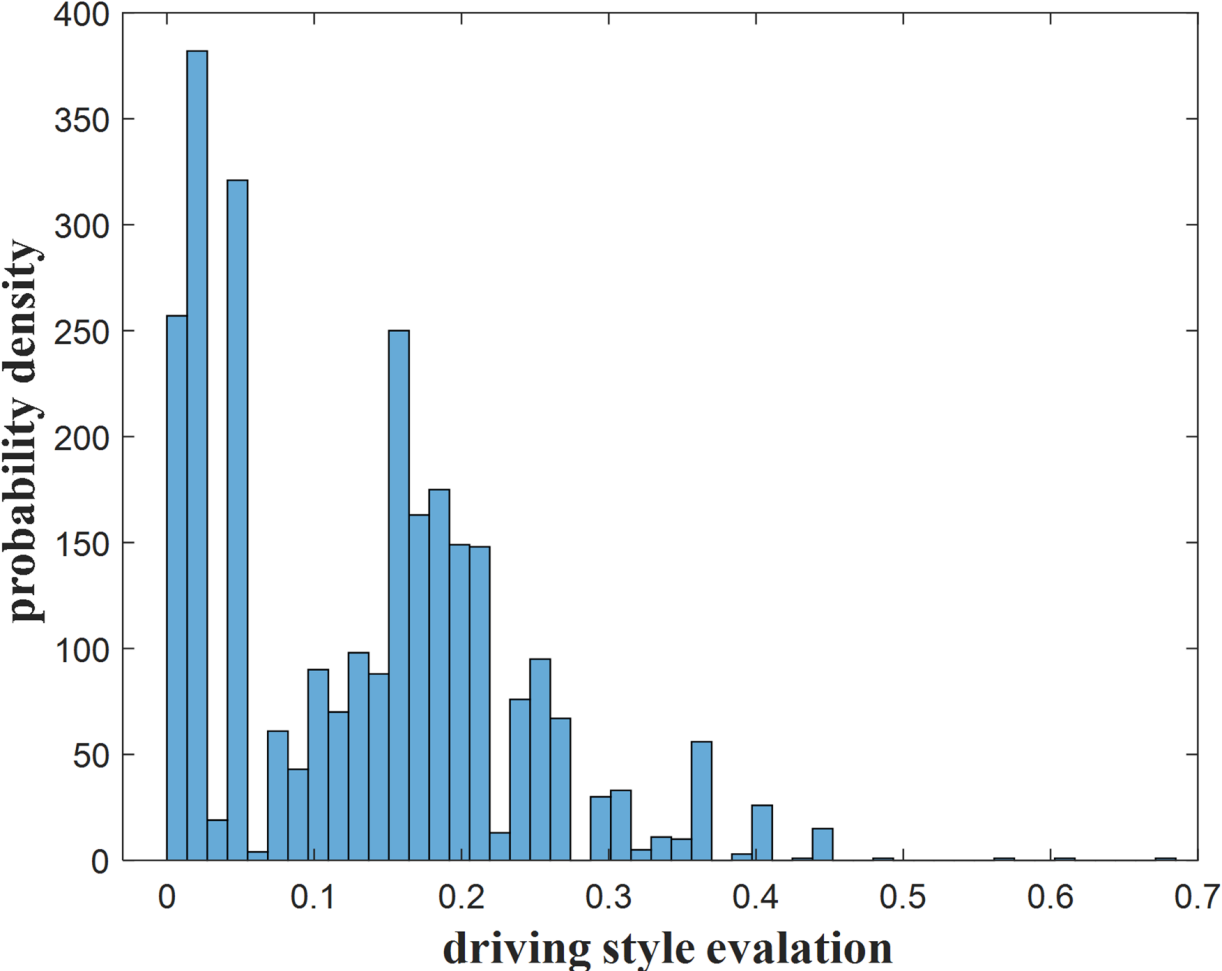
The distribution of driving style score.

Figure 6 presents the distribution of driving style assessment results for 2,763 primitives, with the assessment scores ranging from 0 to 0.6819. The majority of primitives have assessments concentrated between 0 and 0.4. Four primitives, however, have assessments significantly higher than the rest, specifically 0.4824, 0.5648, 0.6066, and 0.6819. A closer examination of these four outliers reveals that the corresponding primitive transitions are 5→4→3, 4→3→4, 3→4→4, and 3→4→1, respectively. Notably, all four outliers involve transitions between the 3rd and 4th primitive categories, which show a marked difference in risk indices between the two categories, with the corresponding transition patterns being relatively rare.

### Style classification thresholds for cautious, normal and aggressive categories

To implement the classification of driving styles and assign labels such as cautious, normal, and aggressive, it is necessary to define appropriate thresholds. Particle Swarm Optimization (PSO) is a nature-inspired optimization technique that simulates the collective behavior of birds during foraging, aiming to find the optimal solution to a given problem. When applied to multi-threshold problems, PSO searches for a set of thresholds that best satisfy the optimization criteria. The optimization criterion typically involves minimizing the within-class variance and maximizing the between-class variance. In this study, the optimization criterion is set to maximize the ratio of between-class variance to within-class variance, as presented in Eq. (8).

8 $$\:P_{{obj}} = N \cdot \:\left( {N_{1} \left( {\mu \:_{1} - \mu \:} \right)^{2} + N_{2} \left( {\mu \:_{2} - \mu \:} \right)^{2} + N_{3} \left( {\mu \:_{3} - \mu \:} \right)^{2} } \right)/\left( {N_{1} s_{1} + N_{2} s_{2} + N_{3} s_{3} } \right)$$

Where *N* is the total number of samples, *µ* is the mean of sample’s style evaluation; *N*_*1*_, *N*_*2*_, and *N*_*3*_ are the sample sizes for the three driving styles, *µ*_*1*_, *µ*_*2*_, and *µ*_*3*_ are the mean style evaluations for the three driving style samples, and *s*_*1*_, *s*_*2*_, and *s*_*3*_ are the corresponding variances.

**Fig. 7:**
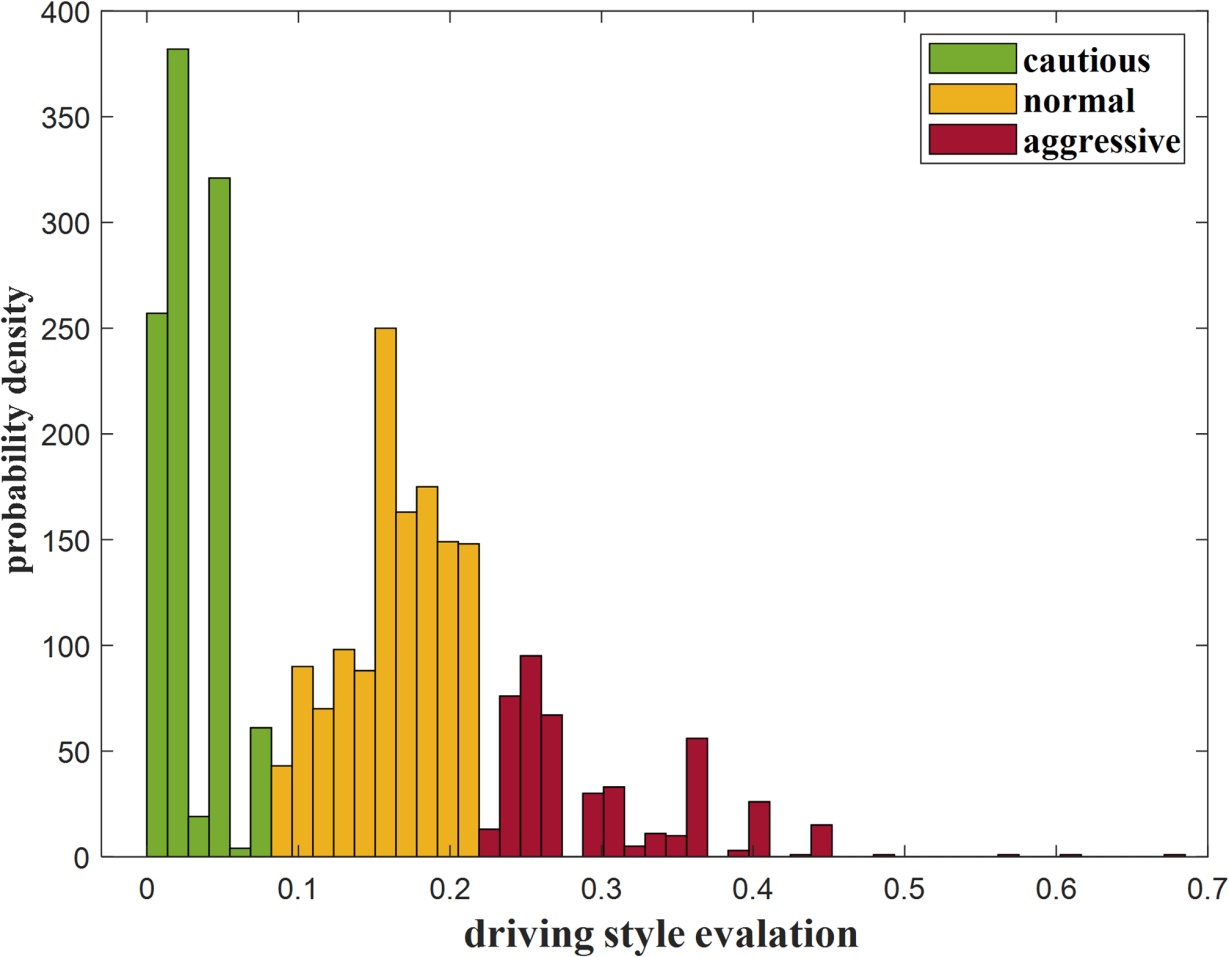
The score ranges corresponding to three driving styles: cautious, normal, and aggressive.

Set the number of particles *P* = 200, particle dimension *d* = 2, and the maximum number of iterations *iter* = 200. The threshold for cautious and normal driving styles is 0.0823, and the threshold for normal and aggressive driving styles is 0.2191. Figure 7 shows the distribution of driving style based on the aforementioned thresholds.

The distribution of the three driving styles shows an approximate peak distribution, with the ratio of cautious: normal: aggressive samples being 1047:1271:445. This result also aligns with the typical distribution pattern of driving styles.

The normal style accounts for the highest proportion, indicating the driver is able to maintain a relatively stable speed and safe following distance for most of the time, demonstrating stable driving behavior. Cautious style samples rank second, indicating the driver adopts more cautious driving maneuvers for a certain amount of time. This driving style is particularly evident in situations involving potential risks and uncertain traffic conditions. The cautious driving style helps to proactively prevent and avoid possible traffic accidents, thereby improving road safety. Aggressive style constitutes only 16% of the overall sample, making it the least common style. This relatively low proportion reflects the significant risks associated with aggressive driving. Such behavior often includes speeding, frequent lane changes, and abrupt braking, all of which can endanger other road users and elevate the likelihood of traffic accidents.

## Discussion

**Fig. 8:**
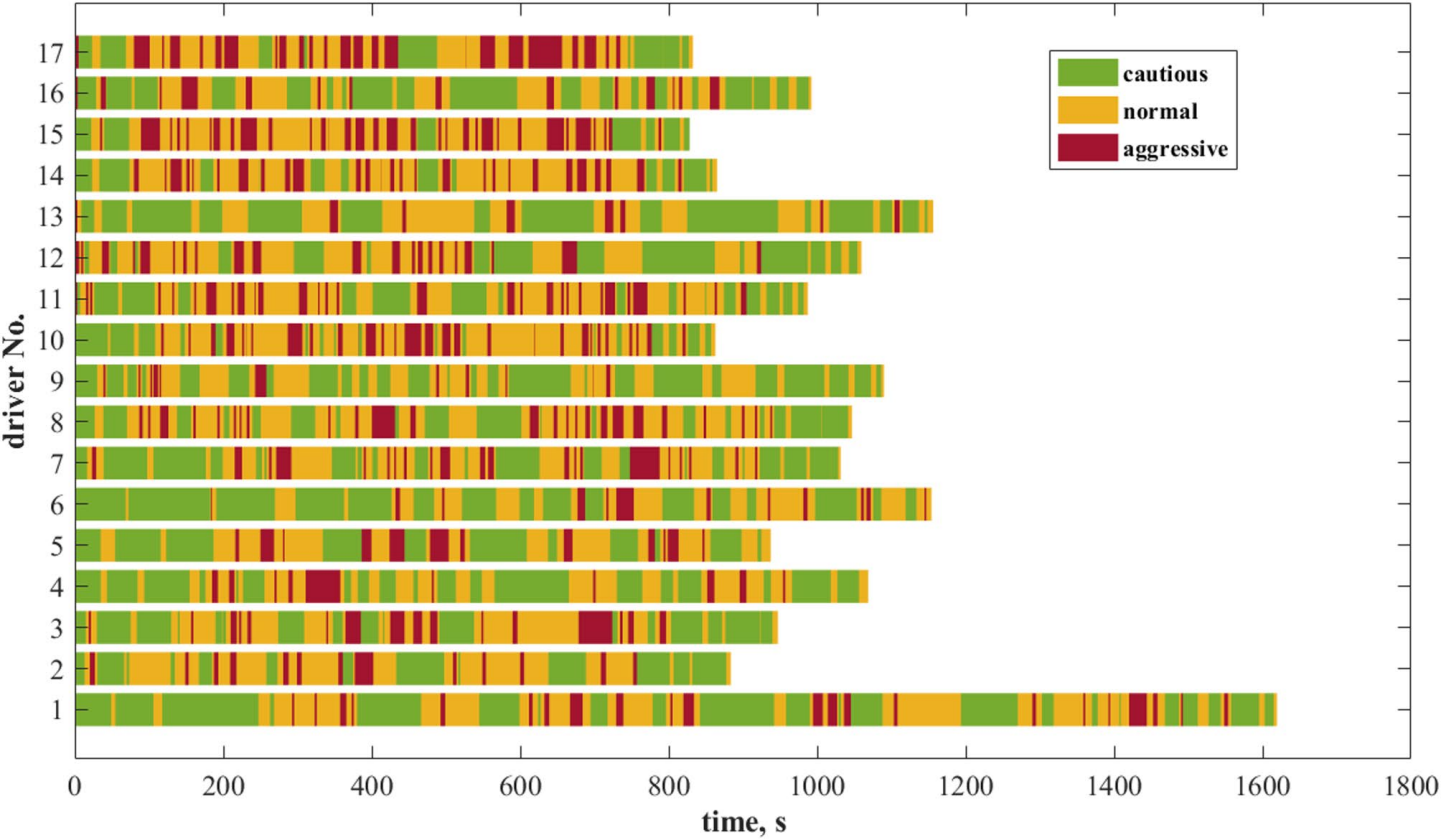
The variation of dynamic driving styles over time for 17 drivers collected in the experiment.

The dynamic driving style sequences for the 17 drivers are illustrated in Fig. 8. The horizontal axis represents time, while the vertical axis indicates the driver number. The driving data were collected in a controlled driving simulator environment. The travel distances for the 17 drivers were consistent, and the traffic conditions during the trips (including surrounding traffic flow and sudden traffic events) were largely identical. Therefore, the dynamic driving style assessment results for these 17 drivers are comparable. Most drivers demonstrate a cautious driving style during the starting and ending phases, with more aggressive and average driving styles observed in the middle segments of the journey. During the starting and ending phases, drivers need to pay close attention to the surrounding environment, which often requires complex driving actions such as acceleration, deceleration, and gear shifting. As a result, they tend to adopt a more cautious driving style. As the journey progresses, drivers gradually adapt to the driving environment. In contrast to the heightened alertness at the start, their psychological stress decreases, which leads to more aggressive driving behaviors and a shift towards a more aggressive driving style.

Also can be seen in Fig. 8, drivers No. 10, 14, 15, and 17 frequently exhibited aggressive driving styles in the middle of the journey, and these four drivers had shorter travel times. In contrast, drivers No. 1, 6, and 13 exhibited aggressive driving styles less frequently, characterized by long intervals of cautious driving between sporadic aggressive behaviors. Furthermore, the travel times for these three drivers were generally longer than those of the other drivers.

To gain a deeper understanding of the relationship between driver behavior and driving style, an analysis is conducted on the driving styles associated with the five primitive types. Table 5 presents the distribution of driving styles corresponding to these five primitive types, where the cautious driving style accounts for a larger proportion in both the 1 st and 3rd primitive type, while the aggressive style is almost absent. This pattern aligns with their lower risk indices, as a lower risk index typically results in a reduced transition risk for these two primitive types. Additionally, Table 3 indicate that, aside from self-transitions, the 1 st primitive type has a higher likelihood of transitioning to the 2nd primitive type, while the 3rd primitive type has a higher probability of transitioning to the 5th primitive type. According to Table 4, the risk indices for these two types of primitive transitions are 0.1328 and 0.0758, respectively, which are considered to be at a medium-low level. As a result, the risk associated with these two transition forms is relatively low as well.

**Table 5:** The distribution of driving styles corresponding to the 2,763 primitives obtained in this study.

The 1 st type	cautious	normal	aggressive
	69.97%	29.26%	0.77%
The 2nd type	0%	70.12%	29.88%
The 3rd type	**89.74%**	9.61%	0.66%
The 4th type	0%	28.42%	**71.58%**
The 5th type	0%	**96.55%**	3.45%

Bold the font to show the probability/proportion that each driving behavior primitive belongs to cautious, moderate, or aggressive driving styles.

The risk indices of the 2nd, 4th, and 5th primitive types are relatively high. Both the 2nd and 5th primitive types are more likely to transition to the 1 st primitive type, while the 4th primitive type is more likely to transition to the 5th primitive type. These transitions contribute to a reduction in primitive risk, with corresponding transition risk indices of 0.0426, 0.0409, and 0.0647, all of which are considered low. Despite their high-risk characteristics, these three types of primitives still result in relatively high driving style evaluation scores. Therefore, the 2nd, 4th, and 5th primitives do not correspond to cautious driving styles. The 2nd and 5th primitives have a higher probability of corresponding to a normal driving style, while the 4th primitive is more likely to lead to an aggressive driving style.

**Fig. 9:**
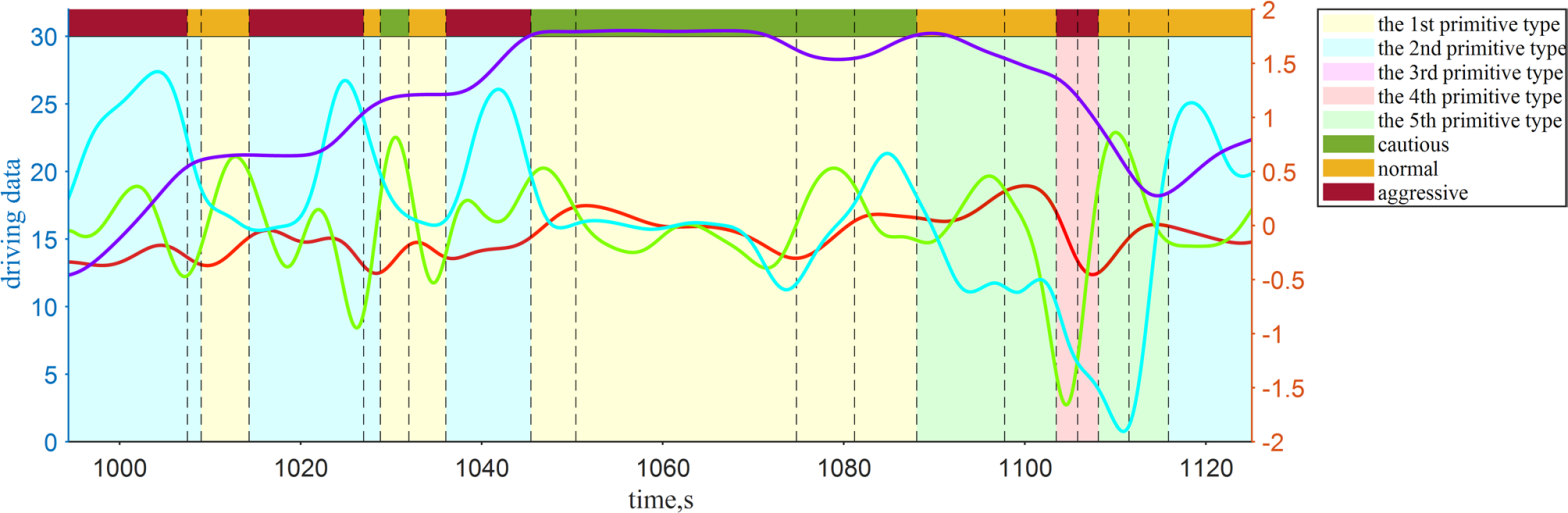
Behavioral primitives within a period of time and their corresponding driving styles.

Figure 9 shows the distribution of behavioral primitives and dynamic driving style for Driver No. 1 during a certain period. In the initial stage, the driver transitions between the 2nd and 1 st primitive types. When transitioning from the 2nd to the 1 st primitive type, both the driving risk and the difficulty of driving operations decrease, and driving style is recognized as shifting from aggressive to normal or cautious. When the 1 st primitive type transitions to itself, the transition risk is 0, resulting in a continuous cautious driving style. In the later stages, the driver transitions between the 5th, 4th, and 2nd primitive types, leading to an increase in driving risk, and the driving style shifts from normal to aggressive.

Although external factors can influence a driver’s behavior, leading to short-term fluctuations in their driving style, each driver typically demonstrates a stable long-term driving style. This long-term style remains relatively consistent and is not easily affected by temporary circumstances. It is deeply ingrained in the driver’s subconscious, shaping their driving actions and decision-making. This style reflects the driver’s habits, risk perception, and safety awareness. Therefore, understanding and identifying a driver’s long-term driving style is essential for developing targeted risk prevention strategies and enhancing driving safety.

**Fig. 10:**
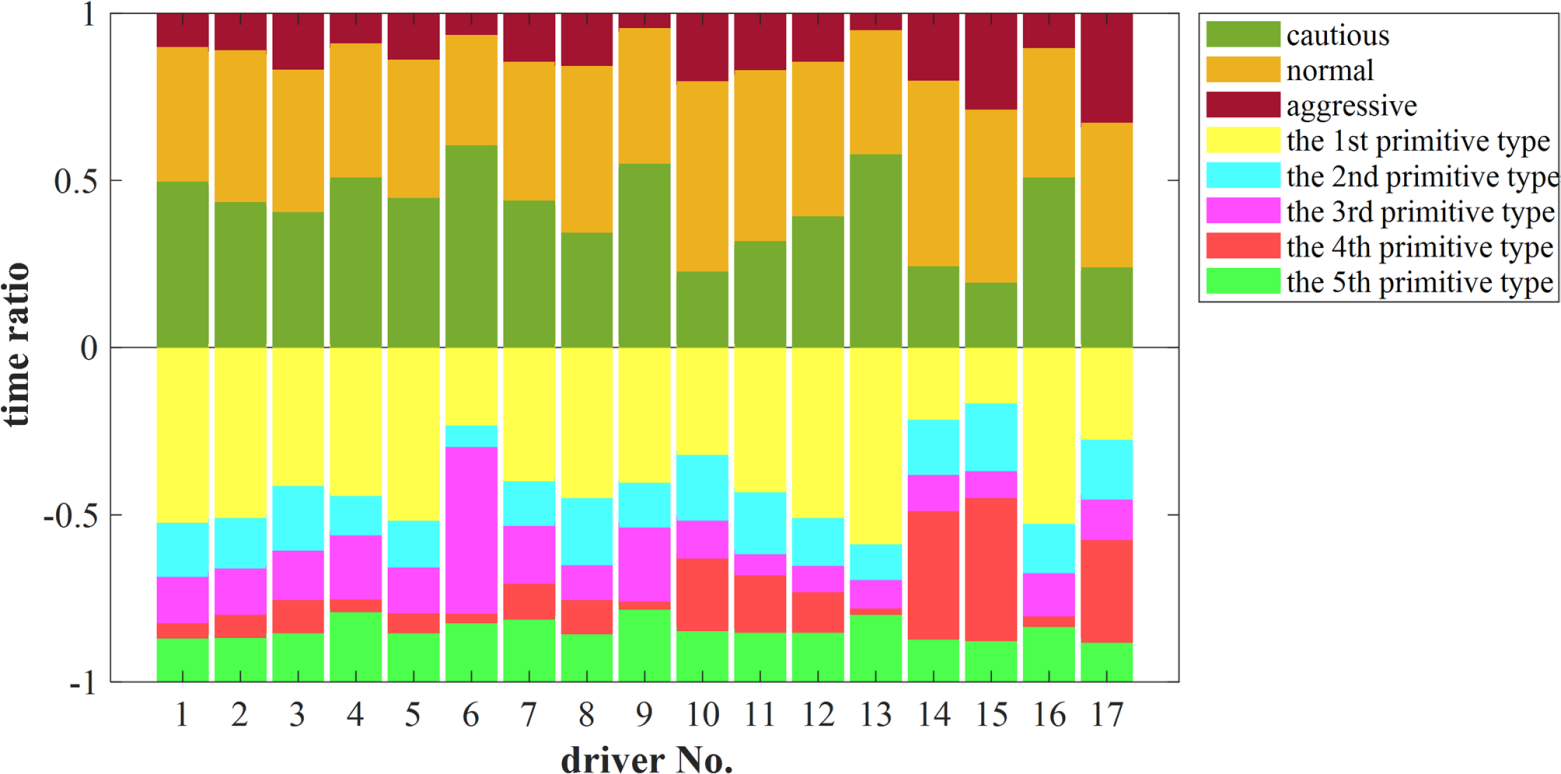
The statistical distribution of driving styles demonstrated by the 17 drivers during a 42 km driving process.

Figure 10 illustrates the distribution of primitive types and dynamic driving styles for the 17 drivers. Drivers No. 10, 14, 15, and 17 exhibit a significantly higher proportion of the 4th and 2nd primitive types, which are associated with high-risk driving behaviors. This indicates a frequent tendency toward aggressive driving, resulting in their long-term driving styles being classified as aggressive. Consistent with the analysis in Fig. 8, these four drivers had relatively shorter travel times. Given the identical mileage, this implies higher average speeds and more intense driving maneuvers. Consequently, from the perspective of intuitive speed observations, the probability of these drivers being classified as having an aggressive driving style is significantly higher.

In contrast, Driver No. 6 possesses the highest proportion of the 3rd primitive type, corresponding to a cautious driving style that persists over time. Similarly, Drivers No. 1, 4, 9, 13, and 16, resembling Driver No. 6, show a higher proportion of the 1 st and 3rd primitive types, which are low-risk, and frequently exhibit a cautious driving style. Combined with the analysis in Fig. 8, these drivers maintained lower average speeds during the experiment and responded to sudden events in a relatively smooth manner. Therefore, intuitively, their driving styles are more likely to be classified as normal or cautious. The remaining seven drivers display a more balanced distribution of primitive types, with their driving styles leaning towards normal, resulting in a long-term classification of normal.

## Conclusions

### The work in this research

Driving behavior primitives are defined as the smallest driving behavior segments with physical significance. They reflect the intrinsic process by which a driver makes decisions and maneuvers in response to the external environment. As fundamental elements, these primitives are crucial for analyzing and understanding driving styles.

In this study, we first extract and cluster primitives from natural driving data collected from 17 drivers, identifying 2,763 valid segments and grouped them into five distinct types: two for steering behaviors and three for straight driving. Using these primitives as the smallest analytical unit, we propose a dynamic driving style evaluation model. This model quantifies driving style by assessing the risk of the current primitive itself alongside the transition risks between adjacent primitives. Specifically, the dynamic style score is derived from a linear weighting of three factors: the risk of the current primitive, the risk of transitioning from the previous to the current primitive, and the risk of transitioning from the current to the next primitive. By applying specific evaluation thresholds for cautious, neutral, and aggressive styles, the model enables the real-time determination of a driver’s dynamic driving style.

The results, validated on the collected dataset, show that the distribution of cautious, neutral, and aggressive styles follows a roughly peak pattern, and the identified dynamic style transitions align with commonly recognized driving patterns. While external factors may induce short-term fluctuations, the model reveals that each driver maintains a relatively stable long-term driving style. By analyzing the relationship between external factors and driver responses at a primitive level, this unsupervised approach significantly enhances evaluation accuracy. This advancement contributes to improved traffic safety, an optimized driving experience, and the development of autonomous driving technologies.

### The future study

#### The enrichment of driving behavior data

The primary focus of this study is to propose a transient and dynamic driving style analysis method based on driving behavior primitives. The core objective of dynamic driving style analysis is to finely reveal the relationship between the external traffic environment and driver responses. While this method can theoretically infer a driver’s long-term, steady-state driving style given sufficient behavioral data, the current study is limited by a relatively small sample size and restricted diversity in driving time, mileage, and environmental exposure. Consequently, the drivers’ specific preferences were not fully exhaustively captured. Therefore, the discussion in Sect. 5 regarding the acquisition of long-term steady-state driving styles should be interpreted primarily as a methodological exploration and validation, rather than a definitive characterization of specific drivers’ long-term habits.

Future work aims to prioritize enriching vehicle data, specifically by extending the driving mileage of individual participants. Building upon the research team’s established research in driver state monitoring, it is planned to develop diversified driving scenarios and collect naturalistic driving data from the same drivers across different times of the day and under various physiological and psychological states. By fusing this data with driving behavior data collected under normal conditions, the study intends to both further validate the proposed methodology and investigate the specific mechanisms by which driver states influence driving styles.

#### The modeling of interaction between the ego vehicle and surrounding vehicles in dynamic driving style analysis

A driver’s driving style is inevitably influenced by the interaction between the ego vehicle and surrounding vehicles. Drivers observe and assess the motion states of surrounding vehicles as well as their relative positions to make behavioral decisions (e.g., lane keeping with deceleration, lane changing) and subsequently control the vehicle’s direction and speed. Consequently, the influence of surrounding vehicles on the ego vehicle is implicitly embedded in the ego vehicle’s operating states. These states essentially represent the outcome of the driver’s decision-making and control processes, based on both the traffic environment and driving expectations (such as safety and efficiency). In light of this, the present study does not explicitly model the motion interaction process between the ego vehicle and surrounding vehicles. Instead, we extract driving behavior primitives based solely on the ego vehicle’s operating states and conduct dynamic driving style analysis exclusively using these states.

Although the proposed method accurately identifies a driver’s dynamic driving style, it does not intuitively reveal the magnitude of the impact exerted by the traffic environment or surrounding vehicles. Furthermore, it is difficult to perform a more detailed analysis of the characteristics and intensity of a driver’s response to unexpected events. Therefore, future research intends to utilize graph-structured data to characterize the motion interaction between the ego vehicle and surrounding vehicles and employ Temporal Graph Neural Networks to mine interaction features. By combining this explicit interaction modeling with the ego vehicle’s driving behavior primitives, we aim to further explore methodologies for dynamic driving style analysis, thereby explicitly uncovering the degree to which surrounding vehicle interactions influence ego vehicle driving behavior.

## Data Availability

The datasets generated during and/or analysed during the current study are available from the corresponding author on reasonable request.
